# Reconsolidation of Traumatic Memories in the Treatment of Complex Post-traumatic Stress Disorder (CPTSD): A Case Study

**DOI:** 10.7759/cureus.68927

**Published:** 2024-09-08

**Authors:** Gunjan Y Trivedi

**Affiliations:** 1 Society for Energy & Emotions, Wellness Space, Ahmedabad, IND

**Keywords:** adverse childhood experiences, anxiety, childhood trauma, complex ptsd, depression, disturbance in self-organization (dso), reconsolidation of traumatic memories (rtm)

## Abstract

This case study explores the memory reconsolidation-based technique of reconsolidation of traumatic memories (RTM) to address complex post-traumatic stress disorder (CPTSD). Using the framework of CPTSD and the components of International Trauma Questionnaire (ITQ), several presenting symptoms and the history of childhood trauma (adverse childhood experiences assessment) were assessed. The individual, based on the trauma-informed care approach, went through a total of eight sessions after the initial consultation that included RTM on index trauma events, reframing, and self-regulation techniques. CPTSD and several internalizing symptoms were measured before and after the intervention. The findings suggest that memory reconsolidation-based RTM protocol, coupled with the constructs of CPTSD using ITQ and index trauma, could provide benefits for individuals with symptoms from prolonged exposure to trauma during childhood.

## Introduction

Mental health assessment involves several subjective components. Specifically, complex post-traumatic stress disorder (CPTSD) represents severe psychological effects from prolonged exposure to several traumatic events during childhood [1,2]. Unfortunately, the Diagnostic and Statistical Manual of Mental Disorders, Fifth Edition (DSM-5) does not officially consider CPTSD as a separate diagnosis. However, ICD-11 has classified CPTSD as a distinct condition separate from PTSD. Research highlights "Disturbance in Self-Organization" (DSO or functional impairment) as the core difference between PTSD and CPTSD [3]. CPTSD significantly influences several areas of daily functioning, covering general tasks, mobility, self-regulation, and interpersonal connections [2,4]. Finally, the evidence also highlights an overlap between CPTSD and borderline personality disorder (BPD) [5,6].

Despite not being included in ICD-10 or DSM-5, recent research on CPTSD has explored its distinct symptoms and treatment approaches, with a specific focus on the impact of repeated childhood trauma (or adverse childhood experiences (ACEs)) [7]. Recent evidence confirms that reconsolidation of traumatic memories (RTM) has emerged as an effective intervention for PTSD [8-11]. With the high prevalence of ACEs and mental health in India, there is a need to explore (a) the assessment of CPTSD and (b) the effectiveness of RTM in addressing CPTSD symptoms using the index trauma framework [11].

## Case presentation

This case study presents the case of a 41-year-old woman, currently a housewife, presenting with extreme fear of losing loved ones. The fear led to high levels of anxiety and depression (highlighted later). She had persistent negative thoughts, relationship issues with her husband, and high anger levels. There was no history of any psychiatric disorders, although she had taken help from a meditation practitioner for persistent negative thoughts (it worked temporarily). There was no history of self-harm but a history of suicidal thoughts (not recently, but at a specific point in time in the past). She highlighted recent deaths and illnesses in the family, which could have made her worry about losing loved ones. She complained about a lack of focus and wanted to eliminate persistent negative thoughts.

Her assessments and current issues highlighted severe levels of anxiety and depression, as shown in Table 1 (GAD-7: Generalized Anxiety Disorder assessment score of 18, MDI: Major Depression Inventory assessment of 31, severe). Her childhood trauma assessment (ACE16 questionnaire, followed by a personal interview) indicated a medium trauma score with a history of (a) frequent emotional abuse, which is linked to a more substantial severity of PTSD symptoms; (b) very frequent physical abuse, especially from teachers; (c) occasional emotional neglect; and (d) mental health issues (and suicide) in the joint family environment where she had grown up [12,13].

**Table 1:** Assessments of WHO-5, MDI, GAD-7, and ISI before and after therapeutic intervention. WHO-5: World Health Organization-Five Well-Being Index; GAD-7: Generalized Anxiety Disorder-7; MDI: Major Depression Inventory; ISI: Insomnia Severity Index.

Timing	Well-being (WHO-5), desired >52	Anxiety (GAD-7), desired >9	Depression (MDI), desired >19	Insomnia (ISI), desired >9
Before consultation	52	18	31	8
After six sessions	56	11	20	2
After eight sessions (completion of therapy)	72	10	9	5
Eight months later	72	6	7	4

While the ACE16 form did not assess the specifics, personal interviews identified the presence of a violent family environment where elders used to have loud fights and emotional abuse. She did not personally experience interpersonal ACEs; however, the home environment had several conditions not usually measured by the ACE assessment template. The personal interview helped identify the challenges not captured in the assessment questionnaires. These included occasional emotional eating, inability to confront the husband for his anger outbursts, and anxiety about losing family members (especially young children and aging parents). There was persistent fear and suppressed anger, indicating an anxious-avoidant attachment coupled with low self-esteem. The interactions with the husband were not satisfactory, given the fear and suppressed anger that prevented the individual from pushing back on simple requests. The loss of elderly family members in the recent past and prolonged illness in the family may have contributed to the fear of losing loved ones. Although the individuals experienced personal attention and love while growing up, the mother was often subject to severe emotional taunts and threats, and this may have contributed to the present assessment of lower perceived social support and loneliness.

Given the history of a violent family environment, history of mental health issues and deaths, high presenting depression levels, and childhood emotional abuse/neglect, a CPTSD assessment was conducted using the International Trauma Questionnaire (ITQ) [2,14,15]. Several index trauma events were identified as the basis for the CPTSD assessment (PTSD score=20, DSO score=20, presence of CPTSD). It is worth adding that a high “Negative Self-Concept” score of 8 contributed significantly to the DSO score, followed by a “Disturbed Relationships” score of 7. Given the high level of resilience and her clear definition of the future state (no negative emotions, positive interactions with family members, and free of the anxiety of losing them), the client agreed to pursue therapeutic intervention.

The intervention spanned six weeks and a total of eight sessions (including two sessions of personalized training for self-assessment that included self-hypnosis, guided imagery, and Emotional Freedom Techniques, excluding consultations). ITQ was administered in the first session, RTM in the second session, followed by the memory reconsolidation process to address the emotional intensity (measured before and after using Subjective Units of Distress (SUD)). The scores for depression, anxiety, insomnia, and well-being were administered after four sessions, after completion of eight therapy sessions, and eight months after therapy completion. CPTSD (ITQ) assessment was repeated after therapy completion and eight months post-therapy completion, yielding the absence of CPTSD. As shown in Table 2, both DSO and PTSD scores reduced significantly. 

**Table 2:** Trauma assessments before and after therapeutic intervention. PTSD: post-traumatic stress disorder; CPTSD: complex PTSD; DSO: disturbance in self-organization; ITQ: International Trauma Questionnaire. PTSD/DSO/CPTSD were assessed using the ITQ.

Timing	CPTSD assessment	PTSD score	DSO score	CPTSD score
Before consultation	Yes	20	20	40
After six sessions	No	4	3	7
Eight months later	No	5	1	6

Several qualitative insights were provided beyond the numbers (SUD for anger decreased from 10/10 to 2/8, and fear of losing a loved one reduced from 10/10 to 6/10). The relationship between the husband and child improved significantly. She said, "I am able to now confront my husband, which was impossible for me to do even for small things earlier." Her self-esteem also increased. Eight months after therapy completion, the individual was in an excellent emotional state, as detailed in Tables 1 and 2. She also expressed gratitude for the intervention and its benefits. Overall, working with individuals who are likely to have CPTSD is not simple, and a trauma-informed care approach is critical. Given that DSM-V is not included in the CPTSD assessment, the capability of the therapist, the ability to avoid re-traumatization, the patience to build trust, and the availability of an alternative therapist to support (if needed) are crucial considerations.

## Discussion

This case study provided a framework to assess the individuals with specific mental health conditions (exposure to childhood trauma, major depressive disorder, severe anxiety, and functional impairment) with the help of ITQ-based CPTSD assessment using the concept of index trauma. Unfortunately, CPTSD assessment, due to its exclusion so far in DSM-V, is not commonly explored in the mental health community [2,16]. The results support addressing CPTSD using index trauma-based identification of the critical events that can be addressed using the RTM protocol in a few sessions. In this specific case, there were two moments of truth. The first was when the individual presented with severe depression (often contributed by low perceived social support and high loneliness, both present) and a high score of negative self-concept (in DSO), the reassurance that science backs what she felt created a possibility that the individual had never believed before [2,7,17]. The second moment of truth was when she could connect the dots using the CPTSD framework with specific events where she was not the center of the events (observed childhood trauma, hostile or violent events around her). The execution (addressing index trauma events using RTM protocol, further identifying irrational decisions in those events where she implicated herself) usually becomes easy [11,15].

It is important to note the linkage between several childhood abuse elements: perceived social support, self-esteem, suppressed anger, and negative self-concept. Usually, the decisions (such as negative self-concept) are extensions of the decisions taken during traumatic events. Hence, the work is required first to identify the index trauma events and address the emotional intensity of those events (using RTM in this case), followed by the process of identifying and addressing irrational decisions. Existing research (evolutionary theory of loneliness, the link between trauma and perceived social support, and long-term risk for CPTSD) supports the insights from this case study [18-20]. 

This case study has a few limitations that must be highlighted. The childhood trauma level was not very severe, and the individual was willing to seek therapy. In our experience, individuals with severe childhood trauma levels and functional impairment are usually reluctant to trust the therapist and need more extended intervention. Moreover, even if such individuals take therapy, they tend to discontinue abruptly. More work is required to assess how consistently such interventions can help in CPTSD.

## Conclusions

This case study highlights the importance of understanding the value of CPTSD assessment, especially in individuals presenting with severe depression, anxiety, low self-esteem, and a history of prolonged trauma exposure during childhood. Given the exclusion of CPTSD assessment in DSM-5, it is likely that such cases need to be assessed using a new framework, such as ITQ. Beyond the assessment, the insights gained from index trauma and memory reconsolidation based on the RTM intervention also add unique value to this case study. Future work should expand the idea to provide more robust insights (larger sample size, diverse population, and rigorous method designs) and the framework.
